# Urinary calprotectin, NGAL, and KIM-1 in the differentiation of primarily inflammatory vs. non-inflammatory stable chronic kidney diseases

**DOI:** 10.1080/0886022X.2021.1885442

**Published:** 2021-03-04

**Authors:** Felix S. Seibert, Maximilian Sitz, Jürgen Passfall, Martin Haesner, Peter Laschinski, Martin Buhl, Frederic Bauer, Benjamin Rohn, Nina Babel, Timm H. Westhoff

**Affiliations:** a Medical Department I, University Hospital Marien Hospital Herne, Ruhr-University of Bochum, Bochum, Germany; b Department of Nephrology, Charité – Campus Benjamin Franklin, Berlin, Germany; c Nierenzentrum Charlottenburg, Berlin, Germany; d KfH-Nierenzentrum Teltowkanalstraße, Berlin, Germany

**Keywords:** Calprotectin, chronic kidney disease, KIM-1, NGAL

## Abstract

**Introduction:**

It has been demonstrated that urinary neutrophil gelatinase-associated lipocalin (NGAL) and calprotectin are helpful biomarkers in the differentiation of intrinsic and prerenal acute kidney injury.

**Objective:**

The present cross-sectional study investigates, whether urinary biomarkers are able to differentiate primarily inflammatory from non-inflammatory entities in chronic kidney disease (CKD).

**Methods:**

Urinary calprotectin, NGAL, and kidney injury molecule-1 (KIM-1) concentrations were assessed in a study population of 143 patients with stable CKD and 29 healthy controls. Stable renal function was defined as an eGFR fluctuation ≤5 ml/min/1.73 m^2^ in the past 12 months. Pyuria, metastatic carcinoma, and renal transplantation were regarded as exclusion criteria. Diabetic nephropathy, hypertensive nephropathy, and polycystic kidney disease were categorized as ‘primarily non-inflammatory renal diseases’ (NIRD), whereas glomerulonephritis and vasculitis were regarded as ‘primarily inflammatory renal diseases’ (IRD).

**Results:**

Urinary calprotectin and NGAL concentrations significantly differed between CKD and healthy controls (*p* < 0.05 each), whereas KIM-1 concentrations did not (*p* = 0.84). The three biomarkers did neither show significant differences in-between the individual entities, nor the two categories of IRD vs. NIRD (calprotectin 155.7 vs. 96.99 ng/ml; NGAL 14 896 vs. 11 977 pg/ml; KIM-1 1388 vs. 1009 pg/ml; *p* > 0.05 each). Albumin exceeds the diagnostic power of the investigated biomarkers by far.

**Conclusions:**

The urinary biomarkers calprotectin, NGAL, and KIM-1 have no diagnostic value in the differentiation of primarily inflammatory vs. non-inflammatory etiologies of CKD.

## Introduction

A kidney biopsy is the only possibility to establish a definite diagnosis of an individual’s renal disease. The histological identification of glomerulonephritis or vasculitis allows the initiation of a specific therapeutic intervention like immunosuppression. In contrast, hypertensive or diabetic nephropathy is primarily managed by unspecific measures like control of blood pressure, diabetes, and hyperlipidemia. Hence, these subjects do not benefit from kidney biopsy. The selection of patients with chronic kidney disease (CKD), who should undergo a kidney biopsy, constitutes an unresolved challenge. Today, the decision to perform a biopsy in a hypertensive or diabetic subject is usually based on clinical scenarios like microscopic hematuria, nephrotic range proteinuria, impairment of renal function with diabetes/hypertension of short duration, or a rapidly worsening glomerular filtration rate (GFR) in previously stable subjects [1]. The definition of these clinical scenarios varies substantially between individual nephrological institutions. It is therefore not surprising that, for example, the rate of reported biopsy-proven coincidental renal diseases in diabetics varies ranging from 3 to 83% [2,3]. There is an unmet clinical need for a diagnostic tool, that allows identification of subjects, that would profit from an individualized immunomodulatory therapy in the setting of stable CKD, hence probably needing a kidney biopsy to reliably determine the disease entity and stage. Unfortunately, there is no biomarker, that reliably differentiates primarily inflammatory kidney diseases from non-inflammatory entities, the latter not suitable for an immunomodulatory medication.

Analogously, there was a search for a biomarker that differentiates between intrinsic and prerenal acute kidney injury (AKI). A reliable noninvasive test for the differentiation of prerenal and intrinsic AKI was desirable since it would shorten the time to initiation of therapy and it would prevent unnecessary biopsies in prerenal disease. We and others have shown that urinary calprotectin and neutrophil gelatinase-associated lipocalin (NGAL) are helpful biomarkers in this context: Whereas urinary concentrations are substantially increased in intrinsic AKI, concentrations are comparable to healthy controls in the case of prerenal AKI [4–9]. A recent meta-analysis summarized the available studies for calprotectin comprising both adult and pediatric populations [10]. Calprotectin is a mediator protein of the innate immune system. It is predominantly released by monocytes and neutrophils and acts as a danger-associated molecular pattern protein (DAMP) [11]. NGAL is secreted from neutrophils and stimulated or damaged epithelial cells of the distal tubule [12]. It exerts a bacteriostatic function of the innate immune system by preventing bacterial iron uptake [13,14]. Kidney injury molecule-1 (KIM-1) is expressed at low levels in the normal kidney as well as in other organs, but its expression is dramatically up-regulated in the kidney post-ischemia/reperfusion injury in proximal tubule cells [15]. In contrast to calprotectin and NGAL, KIM-1 has not been shown to differentiate between intrinsic and prerenal AKI [16]. Conditional expression of KIM-1 in renal epithelial cells leads to progressive interstitial kidney inflammation and fibrosis in rodent models and is therefore supposed to have an unfavorable effect in CKD [17].

Whereas these urinary biomarkers have been extensively studied in AKI, it remains elusive, whether they may provide helpful diagnostic information in stable CKD as well. With regard to their role as mediator proteins of the innate immune system and their substantial increase in intrinsic AKI (calprotectin and NGAL) and the described impact on inflammation and fibrosis (KIM-1), it may be hypothesized that the urinary concentrations of these biomarkers are higher in primarily inflammatory than in primarily non-inflammatory underlying entities of CKD. The present cross-sectional study investigates this hypothesis in a population of stable CKD.

## Methods

### Study design and protocol

The present work constitutes a multicenter study at two nephrological outpatient offices in Berlin, Germany. 143 patients with stable CKD as well as 29 healthy subjects without any medical history serving as control were enrolled. The study population has been described in a former study [18]. CKD was defined according to KDIGO criteria [19]. CKD was regarded ‘stable’ if the glomerular filtration rate (eGFR, MDRD formula) differed ≤5 ml/min per 1.73 m^2^ within the past 12 months. We refrained from including subjects with a higher deterioration of eGFR in order to reliably exclude any states of acute on chronic kidney injury. Exclusion criteria were obstructive uropathy, urothelial carcinoma, metastatic cancer, and pyuria. Etiological entities were categorized in primarily inflammatory renal diseases (glomerulonephritis, vasculitis; group 1, IRD) and primarily non-inflammatory diseases (diabetic nephropathy, hypertensive nephropathy, autosomal-dominant polycystic kidney disease [ADPKD], and ‘others’; group 2, NIRD). This categorization was done despite the fact that inflammatory mechanisms are of relevance in the pathogenesis of these entities as well. The mechanisms, however, can contribute to the progression of the diseases but do not initiate them and do not necessitate immunosuppression. Glomerulonephritis/vasculitis comprised subjects with lupus nephritis, crescentic glomerulonephritis due to polyangiitis with granulomatosis, membranous glomerulonephritis, IgA-nephropathy and mesangioproliferative glomerulonephritis in Schönlein–Henoch purpura, focal segmental glomerulosclerosis, and glomerulonephritis of unknown origin. This study was approved by the local ethics committee of the Charité – University Hospital Berlin (EA4/043/09) and by the ethics committee of the Ruhr-University of Bochum (5019-14). All patients provided written informed consent.

### Measurement of urinary NGAL, KIM-1, calprotectin, and albumin concentrations

Urine samples (10 ml) were collected and stored frozen (−20 °C) until an assessment of biomarker concentrations took place. Urinary concentrations of NGAL and KIM-1 were assessed by ELISA (BPD-KIT-036 from BioPorto Diagnostics and ADI-900-226-0001 from Enzo Life Science, respectively). Urine concentrations of calprotectin were quantified using an enzyme-linked immunosorbent assay (ELISA) kit (PhiCal^®^ Calprotectin, catalog number K 6928, Immundiagnostik AG, Bensheim, Germany) according to the manufacturer’s protocol as published previously [4,5,20]. Albuminuria was documented as the albumin–creatinine ratio (ACR).

### Statistical analysis

Data were analyzed for Gaussian distribution (D’Agostino Pearson). Due to non-Gaussian distribution, data were analyzed non-parametrically. Data are presented as the median and interquartile range (IQR). Numeric data of patients with primarily inflammatory (IRD) vs. non-inflammatory (NIRD) entities were compared using the Mann–Whitney *U* test. An analysis of variance (Kruskal–Wallis) was used to test for significant intergroup differences of numeric parameters between the different etiological entities of CKD. Post-hoc analysis for interindividual comparisons was performed by Dunn’s post-test. Receiver–operating characteristic (ROC) curves were formed in an attempt to determine the accuracy of urinary calprotectin, NGAL, KIM-1, and ACR in the differentiation of primarily inflammatory (glomerulonephritis/vasculitis) from primarily non-inflammatory entities as well as from apparent healthy from patients suffering from CKD. *p* < 0.05 was regarded statistically significant. All statistical analyses were performed using Prism 6 (GraphPad Software, La Jolla, California, USA).

## Results

The study included 143 subjects with stable CKD including 29 female and 114 male patients with a median age of 72 (IQR 67-77). Group 2 (NIRD, *n* = 128, 89.5%) comprised 41 (28.7%) diabetic and 42 (29.4%) hypertensive patients. A combination of diabetes and hypertension as a cause of CKD was postulated in 19 subjects (13.3%). The remaining primarily non-inflammatory etiologies comprised 7 (4.9%) patients with ADPKD and 19 subjects affiliated to ‘others’ (13.3%). 15 (10.5%) patients suffered from glomerulonephritis/vasculitis and are represented in group 1 (IRD). eGFR was higher in IRD (55.4 [IQR 44.3–76] ml/min/1.73 m^2^) compared to NIRD (38.4 [IQR 30.1–46.1] ml/min/1.73 m^2^; *p* = 0.001). A body mass index above 30 kg/m^2^ was seen in 38.8% of the study population. Ferritin (194.2 [IQR 143.0–235.8] vs. 127.0 [IQR 56.0–218.4] ng/ml; *p* = 0.284), hemoglobin (14.5 [IQR 13.7–14.8] vs. 13.4 [IQR 12.2–14.5] g/dl; *p* = 0.104) and C-reactive protein (2.4 [IQR 1.2–5] vs. 2.9 [IQR 1.2–6.8] g/dl; *p* = 0.444) did not differ between the two groups. 29 healthy controls did not suffer from any preexisting overt medical condition. They included 21 male and 8 female subjects with a median age of 74 (IQR 58–77). Assessment of urinary calprotectin, NGAL, and KIM-1 was successful in the whole study population. Biomarker concentrations in dependence on the underlying etiology of CKD are presented in Table 1.

**Table 1.** Concentrations of urinary biomarkers in dependence of the origin of chronic kidney disease.

Etiology of chronic kidney disease	Calprotectin (ng/ml)	NGAL (pg/ml)	KIM-1 (pg/ml)	Albumin/creatinine ratio (mg/g)
Overall CKD population, *n* = 143	98.6 (37.8–425.8)	12 015 (5658–23 448)	1040 (502–2672)	44.2 (9.1–320.5)
Diabetes, *n* = 41 (28.7%)	74.1 (33.0–217.9)	10 414 (5400–27 064)	990 (601–2444)	174.4 (34.7–678.4)
Hypertension, *n* = 42 (29.4%)	137.5 (38.1–562.9)	13 173 (6567–25 936)	1223 (312–3501)	18.1 (8.5–93.1)
Comb. diabetes & hypertension, *n* = 19 (13.3%)	63.3 (24.4–707.8)	18 741 (10 571–35 067)	1246 (623–2680)	29.6 (9–502.2)
Autosomal-dominant polycystic kidney disease, *n* = 7 (4.9%)	50.5 (7.7–404.9)	16 692 (4153–18 534)	1556 (455–2143)	117.2 (6.3–991.9)
Glomerulonephritis/vasculitis (IRD), *n* = 15 (9.8%)	155.7 (20.8–1325.0)	14 896 (5658–28 881)	1388 (833–2730)	198.9 (34.5–1205)
Others, *n* = 19 (13.3%)	144.30 (57.2–448.3)	9706 (3840–14 401)	651 (386–1938)	8.2 (3.6–63.13)
Non-inflammatory renal diseases (NIRD), *n* = 128 (89.5%)	97.0 (39.0–420.7)	11 977 (5658–22 770)	1009 (427–2669)	26.6 (8.8–264.3)
Healthy controls, *n* = 29 (age: 74; 58–77)	56.9 (13.3–124.6)	4765 (2449–9877)	1183 (626–2497)	0 (0–0)
Mann–Whitney *U* test (overall CKD population vs. healthy controls)	**p = 0.0068	***p < 0.001	*p* = 0.84	***p < 0.001
Mann–Whitney *U* test (IRD vs. NIRD)	*p* = 0.81	*p* = 0.41	*p* = 0.34	*p = 0.028

Data presented as median with interquartile range in brackets. CKD: chronic kidney disease; NGAL: neutrophil gelatinase-associated lipocalin; KIM-1: kidney injury molecule 1; NIRD: non-inflammatory renal disease; IRD: inflammatory renal disease. Bold represents statistically significant values (*p* < 0.05). *p < 0.05, **p < 0.01, ***p < 0.001.

### Urinary calprotectin

**Figure 1.**
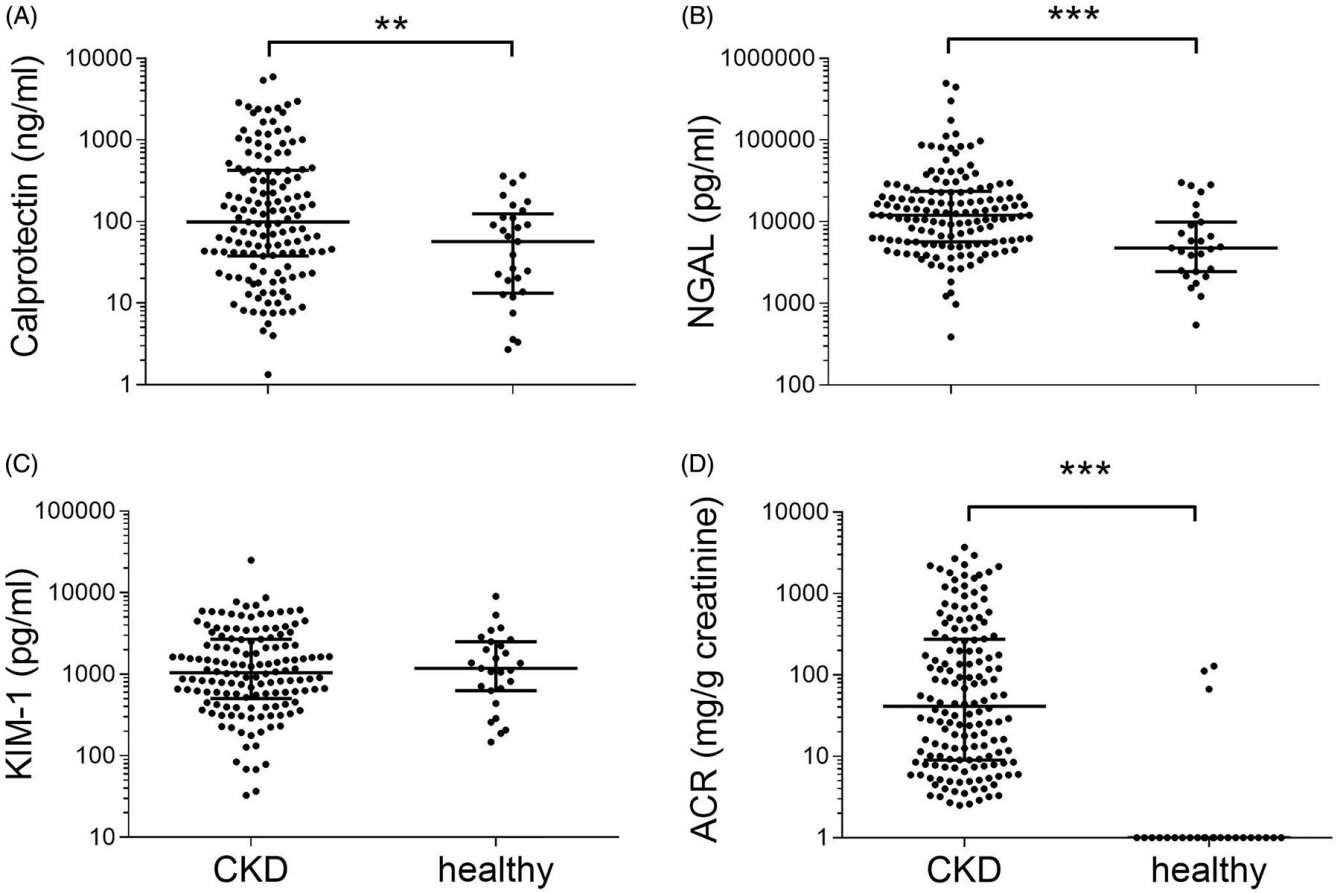
Comparison of the CKD population with healthy controls for urinary (A) calprotectin, (B) NGAL, (C) KIM-1, and (D) ACR. Data are presented as scatter plots (logarithmic *Y*-axis, medians are indicated by horizontal lines). Significant differences were ***p < 0.001 and **p < 0.01 by Mann–Whitney testing. NGAL: Neutrophil gelatinase-associated lipocalin; KIM-1: kidney injury molecule-1; ACR: albumin/creatinine ratio; CKD: chronic kidney disease.

**Figure 2.**
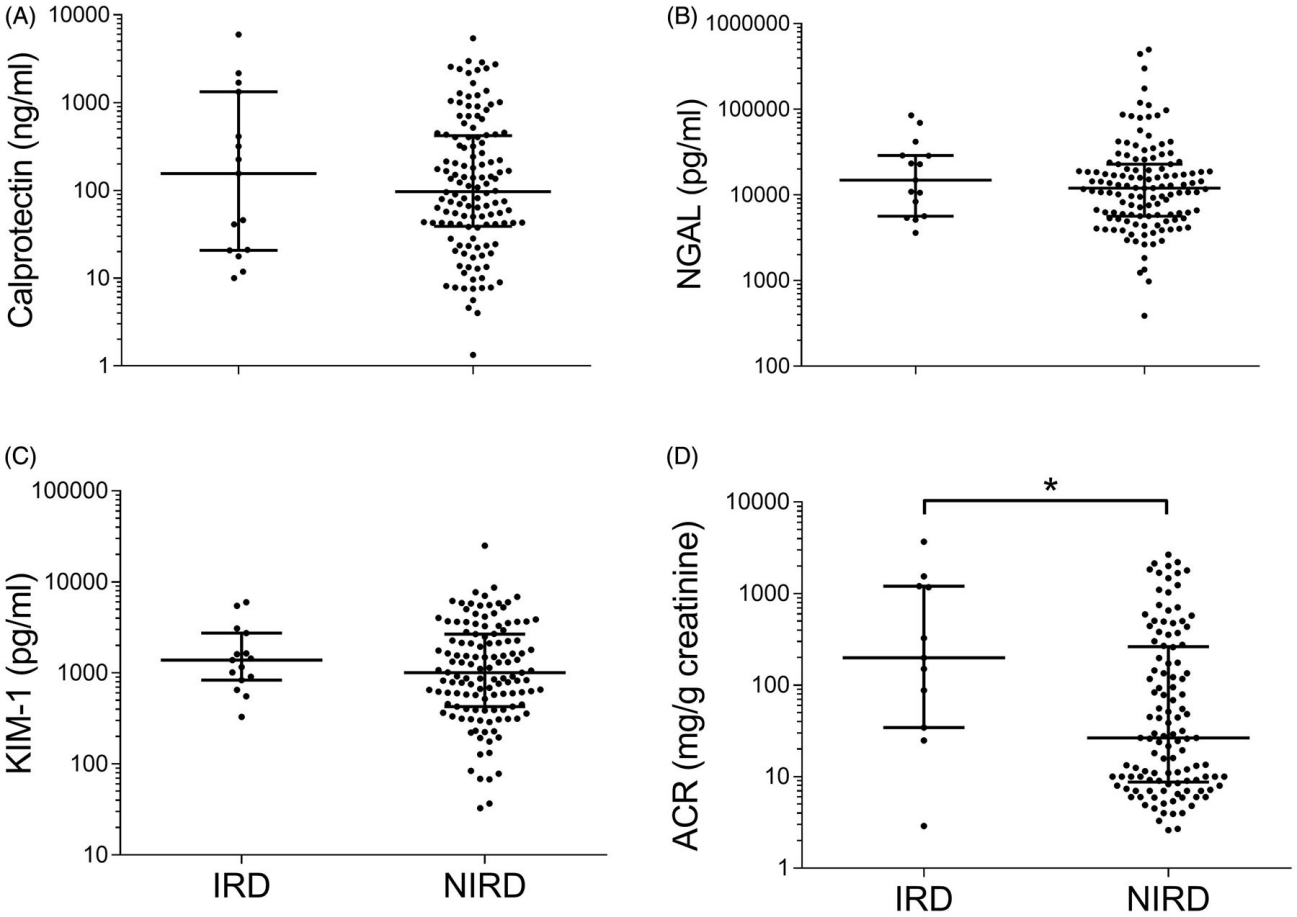
Discriminatory potency of biomarkers between the primarily inflammatory entities glomerulonephritis/vasculitis (IRD) vs. other entities of CKD (NIRD), illustrated by scatter plots (logarithmic *Y*-axis, medians are indicated by horizontal lines) of urinary (A) calprotectin, (B) NGAL, (C) KIM-1, and (D) ACR. Significant differences were *p < 0.05 calculated by Mann–Whitney test. NGAL: neutrophil gelatinase-associated lipocalin; KIM-1: kidney injury molecule-1; ACR: albumin/creatinine ratio.

**Figure 3.**
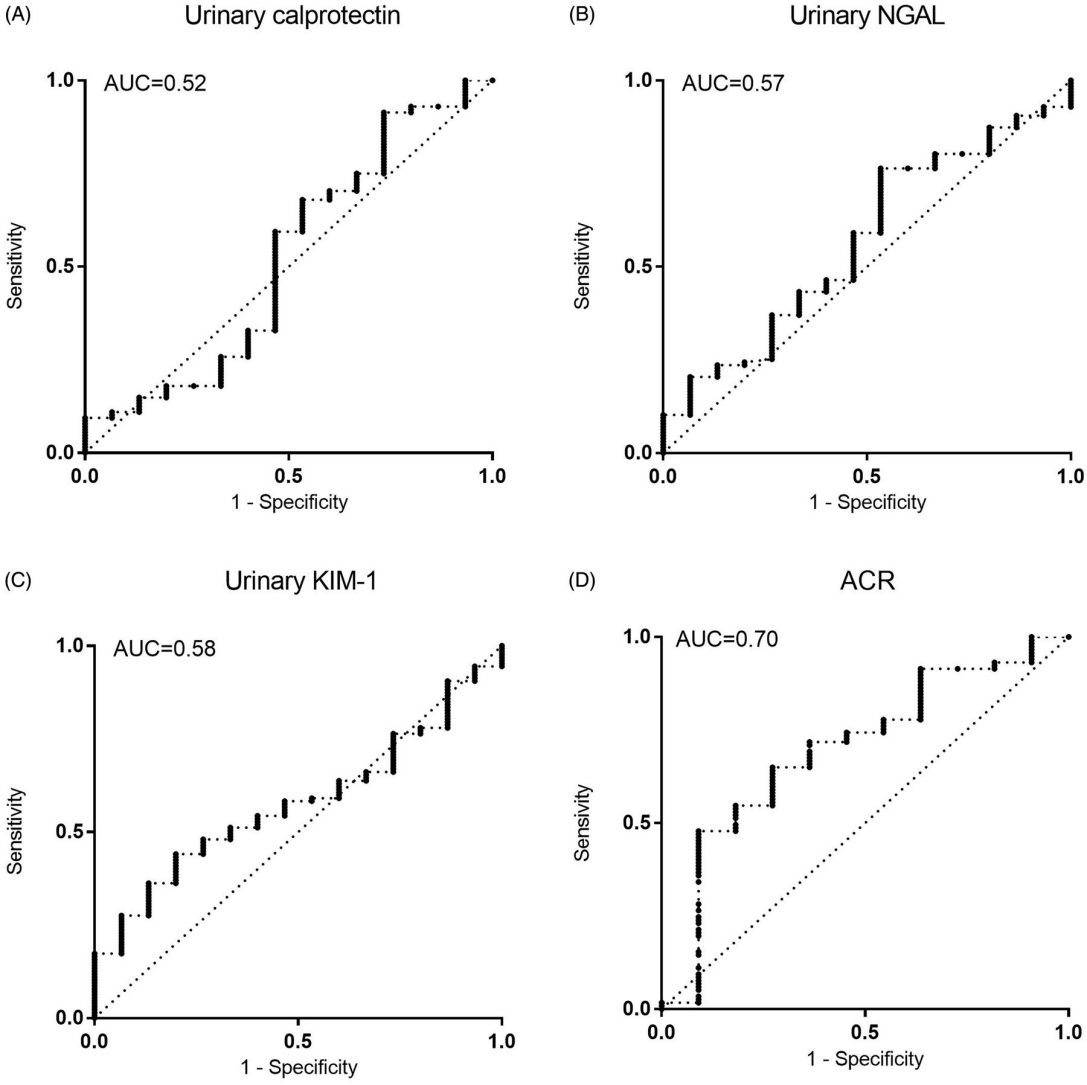
Discriminatory potency of biomarkers between the primarily inflammatory entities glomerulonephritis/vasculitis (IRD) vs. other entities of CKD (NIRD), illustrated by receiver-operating characteristic (ROC) curves of urinary (A) calprotectin, (B) NGAL, (C) KIM-1, and (D) ACR. Diagonal lines indicate differentiation by chance. NGAL: Neutrophil gelatinase-associated lipocalin; KIM-1: kidney injury molecule-1; ACR: albumin/creatinine ratio.

Healthy controls showed significantly lower concentrations (56.9 ng/ml, IQR 13.3–124.6 ng/ml; *n* = 29) than the CKD population (98.6 ng/ml, IQR 37.8–425.8 ng/ml; *p* = 0.0068; Figure 1). The highest concentrations of calprotectin were obtained in IRD. Among NIRD, the lowest concentrations were seen in ADPKD (*n* = 7). Median concentrations of diabetes (*n* = 41), hypertension (*n* = 42), and the combination of these entities (*n* = 19) fell between the values of these two groups. Analysis of variance showed no intergroup differences in the CKD population (*p* = 0.88, Supplements Figure 1). Calprotectin showed a non-significant numerical trend to higher values in IRD compared to NIRD (155.7 ng/ml vs. 97 ng/ml, *p* = 0.81, Figure 2). Receiver–operating characteristic (ROC) curves provided an area under the curve (AUC) of 0.52 in the differentiation of IRD from NIRD (Figure 3).

### Urinary NGAL

NGAL was higher in the CKD population (12 015 pg/ml, IQR 5658–23 448) compared to healthy controls (4765 pg/ml, IQR 2449–9877 pg/ml; *p* < 0.001; Figure 1). The combined etiology of diabetes and arterial hypertension show the highest concentrations (18 741 pg/ml, IQR 10 571–35 067 pg/ml; *n* = 19). Diabetes and hypertension led to median concentrations of 10 414 (IQR 5400–27 064 pg/ml) and 13 173 pg/ml (IQR 6567–25 936 pg/ml), respectively. Subjects of IRD had a median concentration of 14 896 pg/ml (IQR 5658–28 881 pg/ml). Patients with ADPKD yielded a urinary NGAL of 16 692 pg/ml (IQR 4153–18 534 pg/ml). The lowest concentrations were measured in the remaining etiologies of CKD (9706 pg/ml, IQR 3840–14 401 pg/ml; *n* = 19). Analysis of variance showed no intergroup differences in the CKD population (*p* = 0.19, Supplements Figure 1). NGAL showed a numerical trend to higher values in IRD compared to NIRD without reaching statistical significance (14 896 pg/ml vs. 11 977 pg/ml, *p* = 0.41; Figure 2). Receiver-operating characteristic (ROC) curves yielded an AUC of 0.57 in the differentiation of IRD from NIRD (Figure 3).

### Urinary KIM-1

Urinary KIM-1 concentrations did not differ between healthy controls (1183 pg/ml, IQR 626–2497 pg/ml) and the CKD population (1040 pg/ml, IQR 502–2672 pg/ml; *p* = 0.84, Figure 1). ADPKD was associated with the highest concentration of urinary KIM-1 (1556 pg/ml, IQR 454.9–2143 pg/ml; *n* = 7), followed by IRD (1388 pg/ml, IQR 833–2730 pg/ml). The lowest concentrations were obtained in the remaining etiologies (651 pg/ml, IQR 386–1938 pg/ml). Median concentrations of diabetes, hypertension, and the combination of these entities fell between the values of these two groups. Analysis of variance showed no intergroup differences in the CKD population (*p* = 0.77, Supplements Figure 1). KIM-1 showed a numerical trend to higher values in IRD compared to NIRD without reaching statistical significance (1388 vs. 1009 pg/ml, *p* = 0.34, Figure 2). Receiver–operating characteristic (ROC) curves yielded an AUC of 0.58 in the differentiation of IRD from NIRD (Figure 3).

### Albuminuria

Healthy controls (median of 0 mg/g creatinine) had a lower ACR than the CKD population (44.2 mg/g creatinine; *p* < 0.001, Figure 2). The highest ACR was obtained in diabetes (174.4 mg/g creatinine, IQR 34.7–678.4 mg/g creatinine) and IRD (198.9 mg/g creatinine, IQR 34.5–1205 mg/g creatinine). The lowest ratios were measured in hypertension (18.1 mg/g creatinine, IQR 8.5–93.1 mg/g creatinine; *n* = 42) and the combined etiology of diabetes and hypertension (29.60 mg/g creatinine, IQR 9–502.2 mg/g creatinine). Analysis of variance showed an intergroup difference in the CKD population (*p* < 0.001, Supplements Figure 1) with a post-hoc analysis yielding significance for pairwise comparison of diabetes and hypertension (*p* = 0.02) as well as diabetes and ‘others’ (*p* < 0.001). ACR was significantly higher in IRD compared to NIRD (198.9 vs. 26.6 mg/g creatinine, *p* = 0.028, Figure 3). Receiver–operating characteristic (ROC) curves revealed an AUC of 0.70 in the differentiation of IRD from NIRD (Figure 3).

## Discussion

Renal biomarkers have been proven valuable for the differentiation and prediction of adverse outcomes in prerenal and intrinsic AKI [4,5,20,21]. We showed previously, that the prognostic value in stable CKD is neglectable [18]. In the present study, we investigated the diagnostic potential of renal biomarkers in the same heterogenous CKD population regarding the differentiation of primarily inflammatory (IRD) and primarily non-inflammatory renal diseases (NIRD).

The first finding of the study is that concentrations of urinary calprotectin and NGAL were significantly higher in CKD than in healthy controls. KIM-1 concentrations did not differ between these groups. Due to the exclusion of subjects with ‘unstable CKD’ with an eGFR fluctuation of >5 ml/min in the past 12 months, this phenomenon cannot be explained by ‘acute on chronic’ kidney injury, but rather reflects the tubular and/or inflammatory damage and activity of the underlying CKD entity.

The three biomarkers revealed no discriminative potential for the individual etiological entities of the underlying CKD. We decided to test KIM-1 and NGAL in order to reflect proximal and distal tubular injury. Calprotectin as a mediator of the innate immune system was a candidate molecule for the differentiation for IRD and NIRD. Indeed, it showed the highest concentrations in IRD. The discriminatory accuracy as described by the AUC of 0.52 in ROC analysis, however, was far too low to differentiate these primarily inflammatory from the remaining entities. This may be explained on the one hand by its character as a so-called ‘danger associated molecular pattern protein’ (DAMP). It can be released as a proinflammatory danger signal from both leukocytes (predominantly neutrophils and monocytes) and damaged tissue itself [11,22,23]. Primarily NIRD with any kind of damaged structures can evoke a secondary infiltration of leukocytes, for example, in ischemia/reperfusion injury. Thus, calprotectin has been shown to play a pathophysiological role in the regeneration of tubular function after ischemia-reperfusion injury [24]. On the other hand, it is known that immunological mechanisms are involved in the evolution of diabetic and hypertensive kidney disease as well [25–28]. Hence, a completely ‘non-inflammatory’ renal disease does not exist.

KIM-1 and NGAL reflect tubular damage. Hence, these molecules may be candidate biomarkers for ADPKD. Indeed, an increase in urinary KIM-1 reflects tubular damage in the proximal S3 tubule segment [29], and Meijer et al. already demonstrated the correlation of an increase in KIM-1 with kidney volume [30]. Results about NGAL, however, were heterogeneous [30–32]. Indeed, ADPKD patients had the highest urinary KIM-1 concentrations and the second-highest NGAL concentrations. In analogy to calprotectin, there was no relevant discriminatory potential. All three biomarkers do not seem to be suitable for the differentiation of different disease entities in stable CKD, whether it would be primarily an inflammatory entity or renal disease with a predominant tubular injury.

The study did not evaluate novel glomerular biomarkers like podocin or nephrin. It did include, however, albumin as the most established and widespread biomarker of glomerular injury. In fact, the discriminatory potency of ACR was better than NGAL, calprotectin, and KIM-1. There was a significant intergroup difference compared to the aforementioned biomarkers. The highest values were reached amongst the inflammatory entities and diabetes, CKD due to hypertension reached the lowest concentrations. With an AUC of 0.70 albuminuria had the highest discriminatory potency of all the biomarkers in the differentiation of IRD and NIRD.

NGAL, KIM-1, and calprotectin detect acute deteriorations of renal function. There are abundant investigations of NGAL and KIM-1 in various settings of AKI as well as their use as predictors for renal and cardiovascular endpoints, whereas work on its differential diagnostic potential in CKD is extremely scarce. The present study aimed to characterize their diagnostic potency in merely chronic disease. The trial provides information on both a heterogeneous ‘real life’ CKD population and a subgroup of primarily inflammatory renal diseases. Calprotectin was tested for the first time with the aim to discriminate for primarily inflammatory entities. In order to avoid any ‘acute on chronic’ states of kidney disease, we focused on stable CKD with an eGFR loss ≤5 ml in the past 12 months. This aspect in study design was mandatorily for a clear separation of acute from chronic disease. On the other hand, it is a limitation: It cannot be excluded, that there is a diagnostic potency of these biomarkers in progressive CKD. Future studies addressing this question should be welcomed.

## Conclusions

In summary, urinary calprotectin, NGAL, and KIM-1 were not able to differentiate between various entities of stable CKD. Specifically, IRD could not be discriminated from NIRD. Albumin as a traditional glomerular biomarker exceeds the diagnostic performance of these new biomarkers by far. Their characteristics in ‘unstable CKD’, for example, active glomerulonephritis or vasculitis, remain elusive.

## Supplementary Material

Supplemental Material

## Abbreviations

ACR: albumin-creatinine ratio
ADPKD: autosomal-dominant polycystic kidney disease
AKI: acute kidney injury
AUC: area under the curve
CKD: chronic kidney disease
DAMP: danger associated molecular pattern protein
eGFR: estimated glomerular filtration rate
ELISA: enzyme-linked immunosorbent assay
IQR: interquartile range
IRD: inflammatory renal diseases
KIM-1: kidney injury molecule 1
NGAL: neutrophil gelatinase-associated lipocalin
NIRD: non-inflammatory renal diseases
ROC: receiver operating curve
