# Supplementary material for: Urinary calprotectin, NGAL, and KIM-1 in the differentiation of primarily inflammatory vs. non-inflammatory stable chronic kidney diseases
Source: Ren Fail. 2021 Mar 4;43(1):417–24. doi: 10.1080/0886022X.2021.1885442 (PMC7939572; doi:10.1080/0886022X.2021.1885442)
Supplement: Supplemental Material [file IRNF_A_1885442_SM0688.pdf]

Supplements

Supplements Figure 1

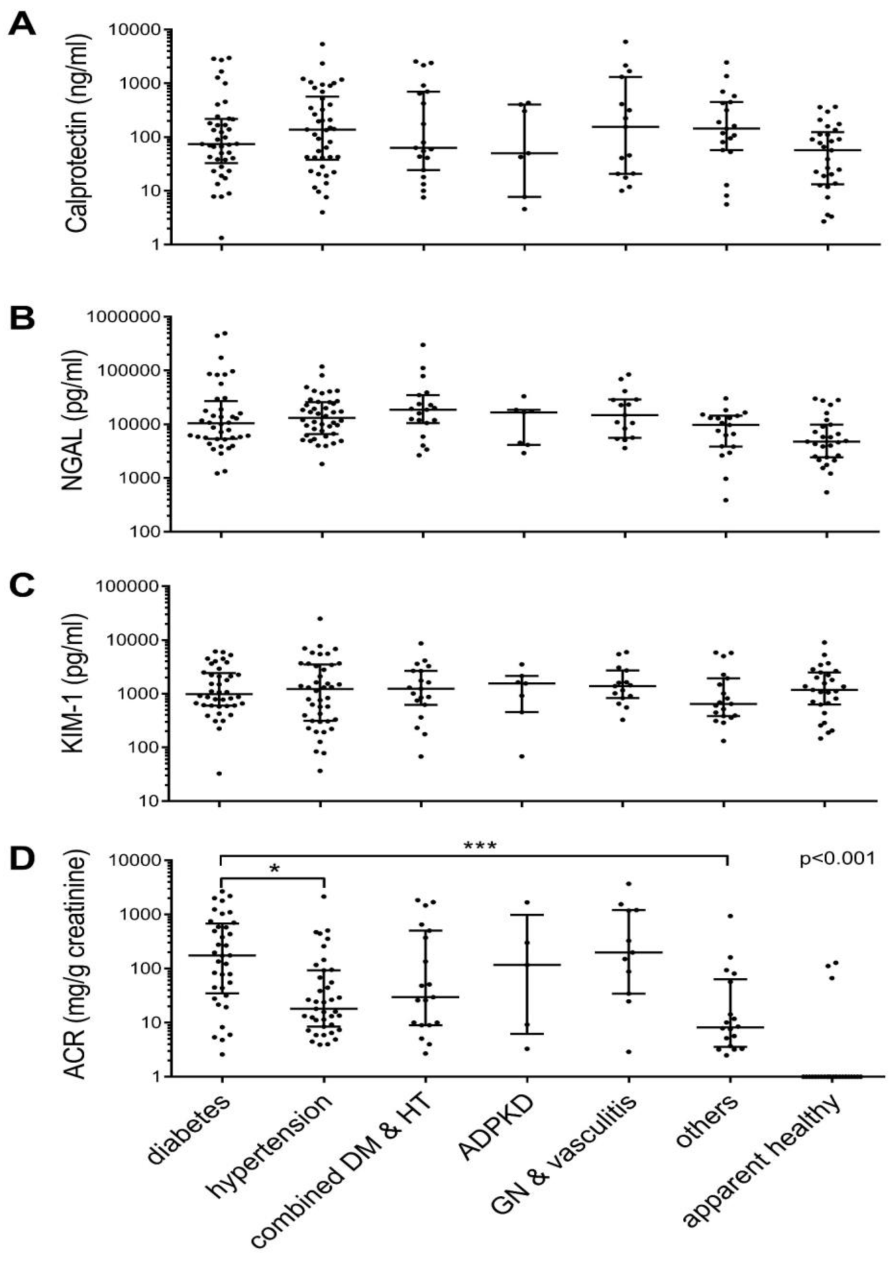

Figure Legend

Figure 1: Individual urinary (A) calprotectin, (B) NGAL, (C) KIM-1 and (D) albumin concentrations in healthy controls and the overall CKD population. Data are presented as scatter plots (logarithmic Y-axis, medians are indicated by horizontal lines). Significant differences between the shown groups were ***P<0.001 and *P<0.05 by Kruskal-Wallis test

and Dunn’s multiple comparison test. DM – diabetes mellitus; HT – hypertension; GN – glomerulonephritis; ADPKD – autosomal-dominant polycystic kidney disease.
